# Editorial: Microbiota-hormones-gut axis as a therapeutic target for metabolic disorders

**DOI:** 10.3389/fendo.2023.1178222

**Published:** 2023-05-18

**Authors:** Anouar Abidi, Kais Rtibi

**Affiliations:** Unit of Functional Physiology and Valorization of Bio-Resources of the Higher Institute of Biotechnology of Beja, University of Jendouba, Beja, Tunisia

**Keywords:** microbiota, gut-hormones, diabetes, thyroid diseases, dyslipidemia

Interactions between the intestinal microbiota, hormones, and the gut itself create a cascade of responses of many types that play an important role in the regulation of metabolic processes. As a result, potential disruptions in this axis will be connected to the development of several metabolic disorders such as obesity, type 2 diabetes and cardiovascular diseases.

Additionally, new efforts to treating these conditions have included directly addressing the microbiota-hormone-gut axis *via* dietary changes, probiotics, prebiotics, antibiotics, and fecal microbiota transplantation.

Dietary changes can have an impact on the composition of the gut microbiota as well as the production of hormones involved in metabolic regulation.

In the same context, Xu et al., revealed the therapeutic effects of a Chinese herbal formula mixed with metformin on the intestinal microbiota of persons with type 2 diabetes. This study found that the therapy combination improved glycemic control while also having a positive effect on the gut microbiota by increasing the abundance of beneficial bacteria such as Bifidobacterium and Lactobacillus while decreasing the abundance of harmful bacteria such as Clostridium and Streptococcus. These findings are consistent with the report from Liu et al., in relation to antidiabetic drugs based on probiotics or prebiotics that alter microbial diversity and composition, as well as levels of bacterial components and derived metabolites, such as lipopolysaccharide (LPS), short chain fatty acids (SCFA), bile acids, and indoles. The latter are known for their importance in the regulation of the intestinal barrier, the inflammatory response, insulin resistance and glucose homeostasis, *via* the intestinal microbiota influencing the secretion of intestinal hormones such as GLP-1 and PYY.

It has also been demonstrated that persons with type 2 diabetes have a distinct makeup of the gut microbiota when compared to healthy individuals, with a diversity and abundance of helpful bacteria reduced and an excess of potentially harmful bacteria increased (1).

Dyslipidemia, another side effect of a metabolic illness, refers to an abnormal level of lipids in the blood, such as cholesterol and triglycerides. It is a prevalent risk factor for major illnesses such atherosclerosis, coronary heart disease, stroke, and other cardio-cerebrovascular disorders… Some recent research, notably Lei et al.’s, have focused on the possible role of the gut microbiota in the development and progression of dyslipidemia. The gut microbiota is important in the metabolism of cholesterol and triglycerides. This study found that changes in the makeup of the intestinal microbiota have a significant impact on lipid metabolism and hence help to prevent the development of dyslipidemia *via* molecular pathways involving short chain fatty acids, bile acids, and trimethylamine N-oxide. They have frequently been associated to increased insulin sensitivity and glycemic management.

On the other hand, the gut microbiota is becoming more recognized as a crucial participant in the development and control of many facets of human health, including the endocrine system. Increasing research shows that the gut microbiota may play a significant role in the development and progression of thyroid diseases. Thyroid diseases, such as thyroid cancer, Hashimoto’s thyroiditis, Graves’ disease, hypothyroidism, Graves’ disease, are characterized by abnormalities in the production and function of thyroid hormones, which can have widespread effects on many aspects of the metabolism and body function. Studies by Jiang et al., suggested that the gut microbiota may influence thyroid function through several mechanisms, including modulating the immune system, affecting hormone signaling, and regulating nutrient uptake and metabolism. These autoimmune thyroid diseases are characterized by the immune system attacking the thyroid gland, leading to inflammation and damage. Moreover, certain intestinal microbiota bacterial species may be implicated in the regulation of thyroid hormone signaling pathways, namely the conversion of thyroxine (T4) to triiodothyronine (T3). Changes in the gut flora can thereby influence thyroid hormone levels and function. Taken together, these studies suggest that the gut microbiota plays an important role in the development and regulation of thyroid function, and that interventions that modulate the gut microbiota may have therapeutic potential in thyroid diseases.

Thus, addressing the microbiota-hormones-gut axis looks to be a viable therapeutic strategy for metabolic disorders and associated diseases. As a result, manipulating the microbiota may be a promising treatment option for many disorders (type 2 diabetes, cardiovascular, dyslipidemia, thyroid, etc.).

## Author contributions

All authors listed have made a substantial, direct, and intellectual contribution to the work and approved it for publication.
